# Sexuality Attitudes and Beliefs Survey (SABS): Validation of the Instrument for the Spanish Nursing Students

**DOI:** 10.3390/healthcare9030294

**Published:** 2021-03-08

**Authors:** Ana Maria Aguiar Frias, Irene Soto-Fernandez, Luís Manuel Mota de Sousa, Sagrario Gómez-Cantarino, Maria da Luz Ferreira Barros, Maria Jesús Bocos-Reglero, Vicki Aaberg, Ermelinda Caldeira, Margarida Sim-Sim

**Affiliations:** 1 Comprehensive Health Research Centre (CHRC) Integrated Researcher, Nursing Department, University of Evora, 7000-811 Evora, Portugal; anafrias@uevora.pt (A.M.A.F.); lmms@uevora.pt (L.M.M.S.); mlb@uevora.pt (M.L.F.B.); ecaldeira@uevora.pt (E.C.); msimsim@uevora.pt (M.S.-S.); 2 School Nurse, Public School, Cuidad de Nara, Avenida Francia 8, 45005 Toledo, Spain; Irene.soto.due@hotmail.com; 3 Faculty of Physiotherapy and Nursing, Campus de Toledo, University of Castilla-La Mancha (UCLM), 45071 Toledo, Spain; Maria.Bocos@uclm.es; 4 School of Health Sciences, Seattle Pacific University, Seattle, WA 98119, USA; aaberv@spu.edu

**Keywords:** sexuality, nurse, student, professor, health education, university, care, holistic

## Abstract

Patient sexuality is a fundamental subject in nursing student’s education. However, beliefs about patient sexuality can influence the care offered. The aim of this psychometric study was to describe the validation process and the psychometrics properties of the Sexuality Attitudes and Beliefs Survey (SABS) for Spanish nursing students. The convenience sample was 283 nursing students from a state university in Spain. Participants ranged from 18 to 30 years (M = 19.66; SD = 1.85). Data collection happened in 2019–2020. The translation, back translation and adaptation to Spanish was discussed and refined, ensuring the semantic, idiomatic and conceptual meaning of the items. The psychometric properties were assessed through analysis of validity and reliability. The Cronbach’s alpha for the final version of 12 items was 0.65. Although it has low reliability, the Spanish version of the SABS seems to be a valid and useful tool to measure nursing students’ beliefs about patient sexuality. In addition, it can be a resource for the assessment of the ability of Spanish nursing faculty in engaging topics involving the student’s vision of patient sexuality.

## 1. Introduction

Human sexuality has been a subject of research since the 1940s, bringing discussion of facts about private life to the public arena. Masters and Johnson, Kinsey and Kaplan are the pioneering figures who first explored and published early studies about sexuality [1,2,3]. Sexuality is observed through behaviors, attitudes and opinions; however, quality tools to make such observations are sparce [4] in several languages, including Spanish. Attitudes are broad concepts which include affect, cognition and behavior [5]. This three-concept focus permits researchers to explore the conflicts that emerge between knowledge, emotions and personal actions and to decide what is valued in a positive or negative way. Sexuality has a deep meaning in one’s personal life and those attitudes can carry over into professional work environments. Health professional students have a lack of knowledge of sexuality and a lack of adequate sexual history taking and sexual counseling skills, which can all result in negative attitudes toward the provision of holistic care for their patients [6]. Students who have received sexuality content during their education and who have interacted with members of the lesbian, gay, bisexual and transgender (LGBT) community are likely to predict more positive attitudes about the provision of sexual healthcare across diverse populations [7]. Health professional student engagement with members of the LGBT community can serve as fertile ground in which to address preconceived ideas about sexuality and lead to lowering barriers to the provision of high quality sexual healthcare.

The development of the Sexual Knowledge and Attitude Test (SKAT), conceived by Miller and Lief in 1979, was the launching pad for research on sexuality [8]. This tool assessed knowledge and attitudes about physical aspects of sexuality and specific sexual behaviors (heterosexual relationships, sexual myths, abortion and masturbation). Two decades of research carrying out the SKAT showed the nursing field as a space were sexuality has not improved over time, and a conservative trend in their sexual attitudes has emerged [9]. In contrast, research with nursing students has been robust in pointing to a higher degree of knowledge and more liberal attitudes in the field of sexuality, compared to nurses [10]. Worldwide, in clinical nursing practice, clear barriers have been identified in the engagement of sexuality. Among these barriers to nurses providing sexual health care, the following stand out: nurses’ beliefs that patients do not expect their sexual life to be addressed, a lack of time to address sexuality in the settings where they provide care, and a lack of confidence and personal comfort in discussing sexuality with patients [11,12,13].

Some studies have identified a lack of research on and discussion of sexuality in the nursing field, both at the academic level and in professional practice [14,15]. The first study that used the Sexuality Attitudes and Beliefs Survey (SABS) carried out with nursing students found that their nursing curricula had not included reference to sexuality in relation to nursing practice. Instead, the students had only analyzed attitudes and knowledge in relation to specific sexual behaviors [15]. Obstacles and limitations to the incorporation of sexuality in nursing education was found. Nursing students need to be required to increase their knowledge about sexuality and develop positive attitudes towards the sexuality topics, and educators need to provide them with essential skills to handle this knowledge [14,16,17].

Sex education in nursing education programs in the United States [18] has shown that sexual education within nursing classrooms is generally deficient and not covered due to higher prioritization of other content and discomfort with the subject. These findings highlight the importance of including sexual competence in nursing curricula as part of educational training to provide holistic patient care [2,18,19]. Understanding care as an integral vision of the person, including their sexual life, is fundamental.

Holistic nursing care is focused on improving health and on respect for all aspects of an individual, which means that moral judgments and personal emotions and reactions have no place in caring [20]. In a review of the use and adaptation of the SABS scale, a study was carried out with 576 nurses from the oncology departments in the United States, in which the same barriers identified previously were shown [21]. Another review of the scientific literature on this topic revealed that few national and international studies address sexuality, its concepts and the integration of this knowledge in the experience of nursing care. For the same authors, the relevance of the research is based on the possibility of generating reflections on sexuality and its concepts in the education of nurses [15,22].

Two European studies have used the SABS to assess nurses’ and nursing students’ attitudes toward patient sexuality. The first study, out of Turkey, with 125 student participants, showed that the use of the SABS was appropriate, and the same barriers as seen in previous studies (lack of time, lack of comfort and lack of confidence) were demonstrated [23]. The second study was carried out with 88 nurses in Sweden and showed that 80% of the nurses identified a lack of time as a barrier to talking about sexual issues, while 60% did not trust their abilities to deal with sexuality issues that their patients were concerned about [24].

In Spain, research shows no existing studies on the validation of the SABS for Spanish-speaking nursing students and that no instrument for evaluating attitudes and beliefs about sexuality in clinical nursing has been created. The objective of this study was to describe the validation process and the psychometric properties of the SABS for Spanish nursing students.

## 2. Materials and Methods

### 2.1. Study Design

This is a psychometric study [25] which constitutes validation of the SABS into Spanish for later use in a multicenter approach. Data collection occurred in the academic year 2019–2020 in a convenience sample. The study has been approved by the Ethics Committee for Scientific Research in the Areas of Human Health and Welfare at the University of Évora (number 18175).

### 2.2. Participants

Nursing students in the first, second and third years of nursing education participated in the study. Inclusion criteria: (1) frequent in regimen full schedule nursing course, (2) have Spanish as maternal language. Exclusion criteria: (1) be in a support program for students with special needs, (2) be in a national or foreign exchange program.

Three hundred and twenty-three students were invited to participate and 283 students opted to participate (87.6% response rate). The ages of participants ranged between 18 and 30 years (mean = 19.66; SD = 1.86). All participants had Spanish as their native language. The sample size is adequate for this kind of validation study, because it responds to the minimum criteria [26] and authors suggest to 200 as regular or 300 as a good sample [27].

### 2.3. Procedures

The study authors requested and were granted permission to use the instrument. All participants provided informed consent. Potential participants were informed that there were no financial or academic incentives to participation. The questionnaire was completed on paper in a classroom environment and participant confidentiality was maintained.

Validation resulted from the verification of: (1) content validity, (2) face validity, (3) reliability, (4) construct validity (through Cronbach’s alpha coefficient) and (5) discriminant validity (through the testing of independent groups). Stability used the split-half test [28].

### 2.4. Measurements

Participants were asked to complete a form that included three sections: (1) demo-graphic characteristics (gender, age, year in school), (2) affective-sexual experiences (whether in a relationship, type of relationship and sexual orientation) and (3) the Sexual Attitudes and Beliefs Survey (SABS).

### 2.5. Data Collection Form

#### Sexual Attitudes and Beliefs Survey

The study tested the reliability and validity of SABS carried out by Reynolds and Magnan [4]. This survey was subsequently used in studies to determine barriers affecting the provision of patients sexual counseling by nurses [8,9].

The SABS is a unidimensional measure made up of 12 items. Participants choose an option on a 6-point Likert-type scale. For each statement in the survey, participants mark the answer that corresponds to their view on a scale of 1 to 6 (1 = strongly agree, 6 = strongly disagree). To avoid trends in responses, seven of the 12 items (items 1, 2, 4, 6, 8, 10, and 12) were scaled in reverse (that is, 1 = strongly disagree, 6 = strongly agree). The total points that can be obtained from the survey will vary between 12 and 72. The higher scores of the total points from the survey reflect the existence of more barriers in the evaluation of sexual problems in patients. The Cronbach’s Alpha value of the original English version of the SABS was found to be 0.75 and 0.82 in two applications.

To obtain semantic equivalence, the survey was translated into Spanish and back-translated to English by experts in English (IS-F; MB-R, respectively), and validated by an English native (VA) and by researchers’ group (AF; MB; LS; SC-C). Content validity was ensured by PhDs in Sexuality and Sexuality Education (MSS; EC, respectively). Face validity was demonstrated in a pre-test with 10 male students and 10 female students: at least two from each year of study. After debriefing, no change was needed. Students’ comments supported the final version of the SABS.

A tentative request to the original author (last on August 2020) asking permission to use SABS has had no response. The study continues following a current idea which admits that a published article is public knowledge [29].

### 2.6. Data Analysis

The data were digitized manually using SPSS 24.0 (Statistical Package of Social Sciences Inc. Chicago, IL, USA). The descriptive statistics are shown in numbers (n) and percentages (%) for the nominal variables and in mean ± standard deviation (X ± SD) for the variables obtained by measurement. To assess the internal consistency of the survey, the reliability coefficient was calculated by Cronbach’s Alpha. The adequacy of the scale to the normal distribution was investigated using a Kolmogorov–Smirnov test. To determine the validity of the survey for Spain, the Mann–Whitney U and Kruskal–Wallis Tests were used to compare the scores on the scale. Values of *p* < 0.05 were considered statistically significant.

The ordering of the importance of the barriers was obtained by sequencing the points of each grade awarded by the nursing students in descending order. Therefore, the barriers with the highest scores were evaluated as the most important.

## 3. Results

### 3.1. Participants Characteristics

The socio-demographic variable shows a more representative female student. The most represented courses are first and second years. Considering the variable sexual orientation, heterosexual is the most representative in the sample (Table 1).

### 3.2. Instrument Results

In the measures of central tendency for each of the 12 elements the highest values were shown in item 6 “*I dedicate time to discuss sexual concerns with my patients*.” (M = 3.68; SD = 1.14) and the lowest was on item 2 “*I understand how my patients’ diseases and treatments might affect their sexuality*” (M = 2.45; SD = 1.08) (Table 2).

### 3.3. Reliability

After doing the necessary reverse of the six items, the pool was observed considering SABS statistics. The item-total correlation varies between −0.017 (item 4) and 0.540 (item 9). Among the 66 inter-item correlations, thirty have r values between 200 and 513 and 36 below r = 200.

The internal consistency of the SABS was demonstrated by a Cronbach’s alpha coefficient of 0.610 (Table 2). However, if item 12 r is kept, the coefficient grows to 0.651, and this was the final decision. Considering the SABS global score in this study with 12 items, the mean of the scale was M = 32.98 (SD = 6.42), with a minimum of 12 and a maximum of 51.

Bipartition was used to assess the stability of the SABS. In the Guttman Split-Half Coefficient determination, a value of 0.717 was obtained, and after application of the Spearman–Brown Coefficient correction formula, it remained at 0.717.

### 3.4. Validity

The instrument was completed in approximately 10 min, suggesting good facial validity and content validity. Before the analysis, the normality of distribution was observed, showing a Kolmogorov–Smirnov of K-S = 0.154 (*p* < 0.001), meaning no normality.

The discriminant validity was observed considering factors like sex (two groups; Mann–Whitney test), year of education (three groups; Kruskal–Wallis test), personal relationship (five groups) and sexual orientation (four groups).

With significant differences, men seem to have more barriers to sexual counseling, once the median is higher (*p =* 0.002). Considering the variable year in course, pairwise comparisons showed significant differences between third course (junior), with lower median toward second (sophomore) or first course (freshman), but not significant between the last two ones. However, the instrument did not find differences considering sexual orientation (*p* = 0.074) (Table 3).

The SABS score has a negative association with age (n = 283; r_s_ = −0.284; *p* < 0.001).

The coefficient of variation (CV) in this sample was 19.5%.

## 4. Discussion

### 4.1. Discuss Participants Characteristics

Participants have similar sociodemographic characteristics in age and gender to nursing students from previous investigations where SABS was applied [11,12]. The time needed to complete the survey tool is consistent with previous studies [13]. The response rate was satisfactory.

### 4.2. Discuss Instrument Characteristics

The time taken to complete, the number of questions and the way in which the questionnaire is presented are fundamental for obtaining data. Although there is no standard for acceptable response rates, the author guidelines put about 75%, 60% and even 50% [26]. In the current study, the response rate is high due to the survey being available in paper format and the able to be provided in the classroom.

The cross-cultural adaptation to language and semantic followed the conventional suggestions in the literature [30,31]. It preserved the intellectual property of the original author citing them [9,29].

The SABS mean in this study was higher than the Portuguese mean (M = 32.98; SD = 6.42 vs. 28.49; SD = 5.24). Applying a *t* test for one-sample, it is possible to see significant differences (*t*_(282)_ = 11.772; *p* < 0.001) between the two. This demonstrates cultural differences in attitudes and beliefs between nursing students from the two countries. Spanish nursing students have more traditional attitudes and beliefs about patient sexuality than Portuguese. This difference is surprising. However, it can be interpreted as being due to a higher representation of men in the Spanish sample (25% compared to 12.2% Portuguese). Such a result perhaps underlines the manifestation of masculine attitudes in a profession dominated by women, as exists in nursing. The age of participants and their sexual formation in terms of orientation and gender identity also influenced the study results. In addition, students are still learning how to function as nurses. A Turkish study revealed the influence of age and sexual formation on SABS results with the statement of a student who felt that the subject of patient sexuality, in his words, is ‘‘too personal…and I am too young’’ [11].

### 4.3. Discuss Reliability

In this study, the SABS Cronbach alpha reliability coefficient was 0.65. This result is lower than the coefficient of the Portuguese study [32], but very near to a similar Turkish sample in senior nursing students [13]. A Cronbach’s alpha value greater than 0.60 can establish internal consistency of the measurements [33], although it is recommended that the Cronbach’s alpha value be greater than 0.70 [13]. This coefficient is sensitive to the sample size and to the small number of items. Besides those aspects, a low alpha was predictable, as the inter-relation between items wasn’t very strong. However, by the standard values of Cronbach’s alpha [28,33], it was still acceptable in this Spanish version of the SABS.

The CV of the mean of SABS of this sample is a good CV = 19.5%, in a range of 10%–20% [34]. When compared with the Portuguese result (CV = 18.4%) [32] this indicates a higher dispersion around the mean of the SABS with Spanish students. This result is perhaps influenced by the larger representation of men in the Spanish sample (25%) than in the Portuguese sample. The same seems to happen in a Turkish study, where CV = 21.1%, in a sample which has 14% of male students [13].

Having no test/retest application, the discussion about stability is centered on the Guttman Split-Half Coefficient determination, which showed an acceptable score. In fact, this coefficient is higher than the Cronbach alpha [28]. This kind of accuracy showed the effect of the items position and it is tenable that, in variables like attitudes or opinions [28] which have a temporal stability, the split-half prevents the memory bias. Truly, no researcher can control these types of bias.

### 4.4. Discuss Validity

The discriminant validity by non-parametric tests tends to differentiate Spanish toward Portuguese. The Spanish version of the SABS discriminates for sex, year of study and personal relationship status. This is in agreement with other studies, in which higher averages are observed in males [11,13]. In fact, female students see sexual health as a more normal subject to address with patients than male students.

It was found that male students who had multiple partners and were in the second year of the course (sophomores) had more barriers to sexual counseling. These results, showing that attitudes become more favorable as students advance in their education, align with other findings [11,13]. The sexual orientation of participants was not revealed to be an influencing factor in the SABS scores. Stigmatizing discourse toward lesbian and gay people proliferates in society and exposes them to homophobia in the workplace [32], and perhaps also when they are in the role of patient. Although the validation process of the Spanish version of the SABS in this study does not differentiate for sexual orientation, these participants are tolerant whatever the sexual orientation of their patients. Literature has demonstrated that nursing students need educational programs regarding knowledge and respect for the LGBT community and individual patients [35]. The various teaching strategies involved in nursing educational programs should aim to improve student knowledge, attitudes and skills related to all LGBT patient health needs [36]. Studies carried out with students from an environment where sexuality is predominantly understood in a confessional or a cultural view [10] and that center on the couple’s relationship or coitus, may justify the differences with the current study.

## 5. Conclusions

The current version of the SABS validated and translated for Spanish-speaking populations, with eleven items, is a valid and reliable tool for evaluating nursing student’s attitudes and beliefs about patient sexuality. It is useful in the context of clinical nursing practice, and has appropriate psychometric properties. It is important to know the beliefs and attitudes about sexuality to promote the development of skills and positive attitudes in nursing students, in order to allow them to address the sexuality and sexual concerns of patients in a holistic manner.

Sex education should be addressed in nursing degree training in order to equip students with the relevant skills to carry out a comprehensive approach to patient care. In addition, it would be advisable for nursing professionals to refresh their knowledge of sexuality in graduate courses to help them in their daily interactions with patients. This would result in greater comfort level and understanding between health professionals and patients, thus creating a stable foundation of supportive and holistic care for hospitalized individuals. The current version of the Spanish language SABS can be an ideal ally to carry out these objectives.

The conclusions presented here differ from those of previous studies, and this may indicate the influence that context can have on attitudes and beliefs about sexuality. For this reason, further studies to reinforce the validity and fidelity of the SABS scale, both with nursing students and with nurses from different cultures and practice contexts, are suggested. Future research, centered on the gender effect of the nursing profession, can eventually be interesting and develop the critical/clinical thinking about attitudes toward patient sexuality.

### 5.1. Limitation

Although the sample size is adequate for this kind of methodological study, the convenience sample hinders an inferential data interpretation. The classroom space where the questionnaires were answered can be a limitation when it comes to the topic of sexuality. A guarantee of anonymous results in terms of which students opted to complete or not complete the study, as could be done in an online format, would be helpful. On the one hand, a larger number of questionnaires was likely returned than with an online option.

### 5.2. Future Research

Although Spain is a parliamentary monarchy, a secular state, it is a Christian country for the most part. Perhaps the variable of religious orientation in the demographic portion of the questionnaire would be helpful to assess the influence of families and the Judeo-Christian tradition on the attitudes and beliefs that students hold about patient sexuality.

Other areas of future research include the assessment of barriers to sexual counseling based on various demographics. As males in this study were shown to have higher SABS scores, does the gender of the nurse and the gender of the patient influence the ability to provide sexuality counseling? Do sexual orientation and gender identity affect the same ability to provide sexual counseling? Are religious beliefs and depth of faith commitment also significant barriers to the provision of sexual healthcare? If the demographic qualities of the nurse and patient are in alignment, is the provision of sexual healthcare more straightforward?

## Figures and Tables

**Table 1:** Characteristics of the sample.

Variable		*n*	%
Sex	Male	71	25.1
	Female	212	74.9
Year in school	1st year	101	35.7
	2nd year	107	37.8
	3rd year	75	26.5
Personal Relationships	One sexual partner	101	35.7
	One sex partner, sex with others	58	20.5
	Multiple sexual partners	24	8.5
	No sexual partner	82	29.0
	Never had a sexual partner	18	6.4
Sexual Orientation	Heterosexual	252	89.0
	Homosexual	12	4.3
	Bisexual	13	4.6
	Not defined	6	2.1
Total		283	100

**Table 2:** Descriptive statistics and internal consistency for the items.

Heading	Mean	Std. Deviation	Corrected Item-Total Correlation	Cronbach’s Alpha if Item Deleted
1. Discutir la sexualidad es esencial para los resultados en salud de los enfermos ^1^ *1. Discussing sexuality is essential to patients’ health outcomes*	2.63	1.21	0.416	0.558
2. Comprendo cómo las enfermedades y los tratamientos de mis pacientes pueden afectar a su sexualidad ^1^ *2. I understand how my patients’ diseases and treatments might* *affect their sexuality*	2.45	1.08	0.455	0.555
3. Me siento incómodo al hablar sobre asuntos sexuales *3. I feel uncomfortable talking about sexual issues.*	2.97	1.34	0.356	0.568
4. Estoy más a gusto hablando con mis pacientes sobre asuntos sexuales que con la mayoría de los enfermeros con los que trabajo ^1^ *4. I am more comfortable talking about sexual issues with my patients than with most of the nurses I work with.*	3.44	1.05	−0.017	0.635
5. La mayoría de los pacientes hospitalizados están demasiado enfermos para interesarse por la sexualidad *5. Most hospitalized patients are too sick to be interested in sexuality.*	3.19	1.23	0.274	0.586
6. Tengo tiempo para discutir con mis pacientes sus preocupaciones sexuales ^1^ *6. I dedicate time to discuss sexual concerns with my patients*.	3.68	1.14	−0.048	0.643
7. Siempre que los pacientes me hacen una pregunta relacionada con la sexualidad, les aconsejo discutir el asunto con su médico *7. Whenever patients ask me a sexuality related question, I advise them to discuss the matter with their physician.*	3.15	1.41	0.309	0.579
8. Confío en mi capacidad para abordar con los pacientes sus preocupaciones sexuales ^1^ *8. I feel confident in my ability to address patients’ sexual concerns.*	2.73	1.17	0.291	0.583
9. La sexualidad es un asunto demasiado privado para discutir con los pacientes *9. Sexuality is a too private issue to discuss with patients*.	2.82	1.48	0.540	0.519
10. Permitir que un paciente hable sobre sus preocupaciones sexuales es una responsabilidad de enfermería ^1^ *10. Giving a patient permission to talk about sexual concerns is a nursing responsibility.*	2.67	1.15	0.322	0.578
11. La sexualidad debería abordarse solo cuando la iniciativa parte del paciente *11. Sexuality should be discussed only if initiated by the patient.*	3.24	1.26	0.326	0.576
12. Los pacientes esperan que los enfermeros les pregunten sobre sus preocupaciones sexuales ^1^ *12. Patients expect nurses to ask about their sexual concerns*.	3.45	1.16	−0.093	0.651

Source: Author’s translation (^1^ reversed items).

**Table 3:** Differences between Variables In Relation To Total SABS Scores.

Variable		Mean Rank	Test
Sex	Male	168.39	^(1)^ U = 5652.5 *p* = 0.002
	Female	133.16	
Year in school	1st year	109.17	^(2)^*X*^2^ = 160.86 *p* < 0.001
	2nd year	213.15	
	3rd year	84.71	
Personal Relationships	One sexual partner	133.37	^(2)^*X*^2^ = 83.593 *p* < 0.0001
	One sex partner, but sex with others	212.92	
	Multiple sexual partners	165.44	
	No sexual partner	105.70	
	Never had a sexual partner	96.03	
Sexual Orientation	Heterosexual	141.70	^(2)^*X*^2^ = 9.324 *p* = 0.074
	Homosexual	179.33	
	Bisexual	145.65	
	Not defined	72.00	

Source: Own elaboration of the authors (^(1)^ Mann–Whitney U test; ^(2)^ Kruskal–Wallis Test).

## Abbreviations

SABS	Sexuality Attitudes and Beliefs Survey
SKAT	Sexual Knowledge and Attitude Test
UCLM	Universidad de Castilla-la Mancha
